# Optic nerve sheath diameter for prediction of intracranial hypertension after ischemic sTrokE – The ONSITE study

**DOI:** 10.1177/23969873251379985

**Published:** 2025-09-29

**Authors:** Philipp Baumgartner, Malin Zahn, Hannah-Lea Handelsmann, Kevin Geier, Sara Petrus, Martin Hänsel, Konstantin Mayr, Theodor Pipping, Andreas R Luft, Lisa Herzog, Susanne Wegener

**Affiliations:** 1 Department of Neurology, University Hospital Zurich and University of Zurich, Switzerland; 2 Cereneo Center for Neurology and Rehabilitation, Vitznau, Switzerland

**Keywords:** Ischemic stroke, ultrasound, optic nerve sheath, optic nerve sheath diameter, malignant media infarction, intracranial pressure, intracranial hypertension, large vessel occlusion stroke

## Abstract

**Background:**

Intracranial hypertension (IH) from brain edema is a life-threatening complication of large vessel occlusion (LVO) stroke, yet clinical monitoring is often unreliable. Non-invasive methods for early IH prediction are needed. This study assessed whether sonographic measurement of the optic nerve sheath diameter (ONSD) could improve the prediction of IH after stroke.

**Patients and methods:**

We prospectively measured the internal optic nerve sheath diameter (ONSDint) via transorbital ultrasound in 65 stroke patients and 30 controls. ONSD was also measured on the initial CT or MRI. The primary endpoint of IH was a composite of clinical and radiological signs of brain swelling. A predictive ONSD cut-off was determined from a multivariable logistic regression model, adjusted for age and infarct volume. Predictive performance was assessed using leave-one-out cross-validation.

**Results:**

Seven of 65 stroke patients (11%) developed IH. The initial sonographic ONSDint was significantly increased in patients who developed IH. The multivariable model identified an optimal predictive cut-off of ⩾5.51 mm, which predicted IH with a sensitivity of 85.7% and a specificity of 94.8%. In comparison, ONSD derived from initial neuroimaging was also a strong predictor, with an optimal cut-off of 6.80 mm yielding a sensitivity of 100% and a specificity of 91.1%, and showed superior predictive accuracy in the cross-validation (AUC 0.905 vs 0.687).

**Discussion:**

Our sonographic ONSDint cut-off of ≥5.51 mm aligns well with recent stroke literature that used similar standardized measurement techniques. Our findings also highlight the distinct roles of different imaging modalities. While the initial CT/MRI provides a static measurement with high predictive power, the unique advantage of sonography is its bedside applicability, allowing for the crucial, non-invasive serial monitoring of ONSD as a dynamic marker of intracranial pressure changes.

**Conclusion:**

Early ONSD assessment is a valuable predictor of IH after severe stroke. A sonographic ONSDint of ⩾5.51 mm identifies patients at high risk with excellent accuracy. While initial neuroimaging may offer superior predictive power, bedside sonography remains a crucial, repeatable tool for monitoring these critically ill patients.

## Graphical abstract

**Graphical abstract:**
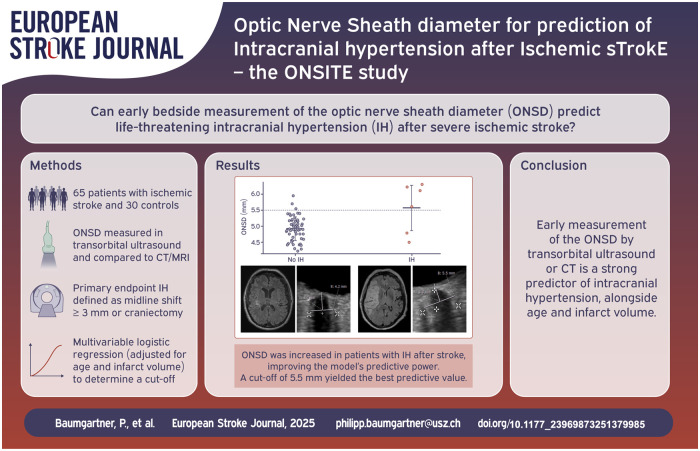

## Introduction

Intracranial hypertension (IH) is a life-threatening complication of ischemic stroke. It occurs primarily in stroke due to large-vessel occlusion (LVO), particularly in large infarcts with occlusion of the internal carotid artery (ICA) or the proximal segment (M1) of the middle cerebral artery (MCA). Without recanalization, up to 30% of patients develop a malignant infarction with large space-occupying brain edema and neurological deterioration.^1,2^ IH usually develops within the first 24–96 h after stroke; however, the exact time course is unpredictable.^3–6^ IH demands immediate medical or surgical action. Therefore, it is necessary to monitor patients at risk closely. However, clinical observation of neurological symptoms may be hampered by pain or anxiolytic medications, infection, cardiac diseases, or delirium. Despite these limitations, intracranial pressure (ICP) is not routinely monitored in stroke patients, because gold-standard ICP monitoring is invasive.^5,7,8^ Serial computer tomography (CT) or magnetic resonance imaging (MRI) are often applied to detect signs of increased ICP in patients at risk. However, imaging is expensive, time-consuming and potentially stressful for the patients.^9^

**Figure 1.**
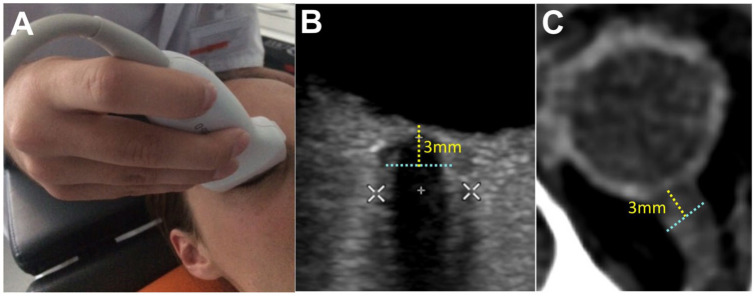
Standardized Measurement of the Optic Nerve Sheath Diameter (ONSD): (a) application of the high-frequency linear probe for transorbital sonography, (b) ultrasound image demonstrating the measurement of the internal ONSD (ONSDint, light blue dashed line) 3 mm posterior to the globe. The white calipers indicate the measurement of the external ONSD, and (c) corresponding ONSD measurement on an axial CT scan.

Ultrasonography is an alternative tool to measure ICP non-invasively. Ultrasonography is a low cost, simple bedside tool, widely available in emergency rooms as well as on stroke units.^10^ Transorbital Sonography (TOS) to measure the optic nerve sheath diameter (ONSD) has been developed and suggested as an alternative sonographic method to detect IH.^9,11–13^ It utilizes the fact that the optic nerve sheath, which is readily visualized by transorbital ultrasound, is filled with spinal fluid and grows in diameter according to ICP (Figure 1).^14^ The utility of ONSD for detecting raised ICP is well-established, with a key meta-analysis demonstrating high sensitivity (90%) and specificity (85%) in patients with acute brain injury.^9^ More recent reviews continue to support its role as a valuable monitoring tool across a range of non-traumatic brain injuries, including ischemic stroke.^15^ Reinforcing its clinical potential, a recent large multi-center study has confirmed that sonographic ONSD is a strong, independent predictor for mortality in patients with large hemispheric infarction.^16^ Currently, TOSis not used in patients with stroke in clinical routine. The goal of this study was to assess whether sonographic measurement of ONSD can be used to predict the development of brain edema and IH after ischemic stroke. We applied repetitive ONSD measurements up to 120 h after symptom onset to find out if there is an optimal time window for ONSD to predict IH.

## Methods

### Study design

ONSITE was a single-center prospective observational study performed at the stroke center of the University Hospital Zurich. The study was approved by the cantonal ethics committee Zurich (BASEC-Nr: 2018-01946) and is registered at ClinicalTrials.gov (NCT06939114). The study was conducted in accordance with the Declaration of Helsinki and is reported following the STROBE statement. Participants were recruited according to the guidelines for research in emergency situations of the Swiss Ethics Committee. Consent was sought from participants or their legally authorized representatives. If unobtainable due to the emergency, the patient was included and initial research procedures were initiated following approval by an independent physician, with deferred consent obtained from the participant or their representative at the earliest opportunity.^17^

### Study population

We prospectively enrolled two cohorts: patients with acute ischemic stroke (AIS) and a control group of non-stroke, non-IH patients.

Stroke patients were eligible if they were ⩾18 years of age, presented with an anterior circulation stroke, and were last-seen-normal within 24 h. The control group consisted of age-matched patients without acute neurological deficits to establish reference ONSD values.

Exclusion criteria for both groups included known intracranial space-occupying lesions, a prior history of IH, or conditions precluding reliable follow-up or informed consent (e.g. severe dementia, active substance abuse).

### Study procedures

#### Clinical variables

For all included patients, baseline data were collected from the electronic medical records. This included demographics, medical history, and cardiovascular risk factors. Clinical data, including the National Institutes of Health Stroke Scale (NIHSS), Glasgow Coma Scale (GCS), and modified Rankin Scale (mRS), were assessed at baseline and during follow-up.

#### Ultrasound measurements

Transorbital sonography (TOS) was performed at up to seven time points (0–12, 12–24, 24–36, 36–48, 48–72, and 72–120 h after stroke onset). Missed time periods due to early discharge or patient transfer were not repeated, but measurements were resumed at the next scheduled interval. To be included in the final analysis, patients had to have at least one ONSD measurement within the first 24 h after stroke onset.

All measurements were performed using a Siemens Acuson or Philipps EPIQ 5 device with a high-frequency linear probe (12–18 Hz). For each eye, multiple transverse views of the optic nerve were obtained to select the single image with the clearest anatomical differentiation for measurement.

In accordance with recent consensus criteria,^18^ the internal optic nerve sheath diameter (ONSDint) was measured 3 mm posterior to the globe. This was defined as the distance between the outer edges of the hyperechogenic band of the optic nerve sheath, explicitly excluding the outer hypoechoic dura mater (Figure 1). Images were controlled for potential technical and imaging artifacts as described by Hirzallah et al.^19^ To minimize potential assessment bias, all images were analyzed in a randomized order with the investigator blinded to patient outcomes and all previous measurements.

#### Neuroimaging measurements

All patients underwent routine clinical neuroimaging. An expert, blinded to all sonographic data and clinical outcomes, analyzed the scans. On the initial CT or MRI scan (obtained within 24 h of symptom onset), the ONSD was measured 3 mm posterior to the optic globe on axial slices of CT or MR angiography sequences, using an electronic caliper (DeepUnity Review, Version 1.1.1.1; Figure 1).

Final infarct volume was determined by manual segmentation of the lesion on diffusion-weighted MRI, preferentially from the 24-h scan, or on follow-up CT imaging (syngo.via, Siemens Healthcare). Additionally, the degree of brain edema was descriptively classified as none, mild-to-moderate (sulcal effacement or ventricular compression), or severe (midline shift ⩾ 3 mm).

#### Primary endpoint

The primary endpoint of intracranial hypertension (IH) was defined as either a midline shift of ⩾3 mm on follow-up imaging, an otherwise unexplained decrease in the Glasgow Coma Scale (GCS) score, or the clinical need for decompressive hemicraniectomy.

#### Statistical analysis

Continuous variables were reported as median ± interquartile range (IQR), categorial variables as frequencies and percentages. Continuous variables were reported as median and while categorical variables were presented as frequencies and percentages. An independent sample *t*-test was used to compare baseline ONSD between stroke patients and controls.

To predict IH, we developed two multivariable logistic regression models. The baseline model included age and infarct volume as predictors. The variables included were chosen based on previous literature.^20^ The second, full model additionally included the initial sonographic ONSDint value. The improvement in model fit was assessed with a likelihood-ratio test.

An optimal ONSDint cut-off value for predicting IH was determined from the full multivariable model using Youden’s Index on the receiver operating characteristic (ROC) curve. The predictive performance of the models was externally validated using leave-one-out cross-validation (LOOCV), and the area under the curve (AUC) was calculated. All analyses were performed using R (Version 4.4.1).

## Results

**Figure 2.**
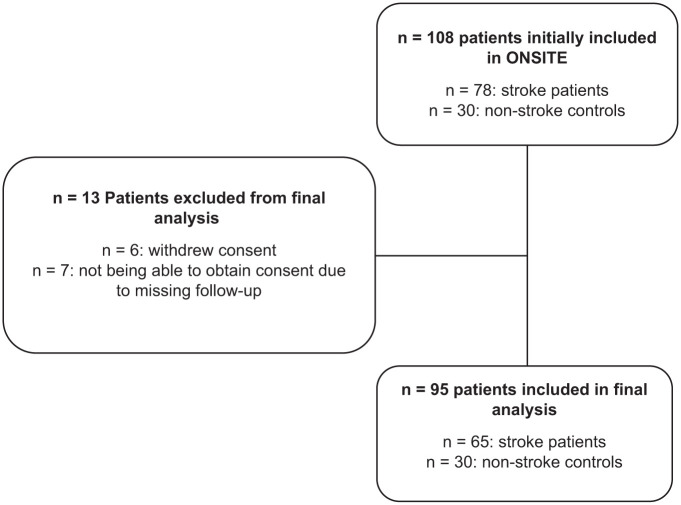
Flow chart of included and excluded patients.

Between January 2019 and January 2022, we prospectively enrolled 78 patients with acute ischemic stroke and 30 control subjects. After exclusions (detailed in Figure 2), the final analysis included 65 stroke patients for the primary sonographic analysis and 30 control subjects. Baseline characteristics for both groups are presented in Table 1. As controls were recruited from the general neurology ward or outpatient unit, their main diagnoses were Parkinson’s disease, TIA, and cerebral atherosclerosis (Supplemental Table 1). In the stroke cohort, 72% of patients had suffered a large vessel occlusion (LVO), most frequently of the MCA-M1 segment. The majority received acute recanalization treatment, and successful recanalization (TICI 2b/3) was achieved in 96% of LVO cases.

**Table 1.** Summary of baseline characteristics of all included patients.

Characteristic	Stroke *n* = 65	Controls *n* = 30
Age, median (IQR)	70 (16)	71 (16)
Sex, female (%)	26 (40)	10 (33)
BMI, median (IQR)	25 (6)	26 (5)
Atrial fibrillation (%)	18 (28)	4 (13)
Diabetes (%)	9 (14)	2 (7)
Arterial hypertension (%)	38 (58)	11 (37)
Dyslipidemia (%)	14 (22)	4 (13)
Current smoker (%)	12 (18)	7 (23)
Prior ischemic stroke (%)	8 (12)	7 (23)
NIHSS on admission, median (IQR)	9 (11)	—
GCS on admission median (IQR)	14 (3)	—
Large vessel occlusion (%)	47 (72)	—
Type of occlusion		
ICA (%)	13 (20)	—
MCA-M1 (%)	28 (43)	—
Tandem occlusion (%)	6 (9)	—
Acute treatment		
IVT only (%)	11 (17)	—
EVT only (%)	25 (38)	—
Combination (%)	22 (34)	—
Type of edema developed		
None	47 (72)	—
Mild-to-moderate edema	10 (15)	—
Severe edema	7 (11)	—
Hemicraniectomy (%)	2 (3)	—

BMI: body mass index; NIHSS: National Institutes of Health Stroke Scale; GCS: Glasgow Coma Scale; ICA: internal carotid artery; MCA-M1: middle cerebral artery M1 segment; IVT: intravenous thrombolysis; EVT: endovascular treatment.

In the stroke group, 19 patients (29%) developed some degree of brain edema, with seven patients (11%) meeting the primary endpoint of IH. The median time from stroke onset to reaching the endpoint was 25.5 (IQR 15) h. The subset of patients developing IH were younger, had less cardiovascular risk factors and had a lower rate of prior strokes compared to the non-IH group (Supplemental Table 2).

### TOS in non-stroke, non-IH controls

In the 30 control subjects, the mean ONSDint, calculated as the average of all measurements per patient, was 4.66 ± 0.46 mm (Supplemental Figure 1).

### Longitudinal ONSDint measurements and comparison between eyes

**Figure 3.**
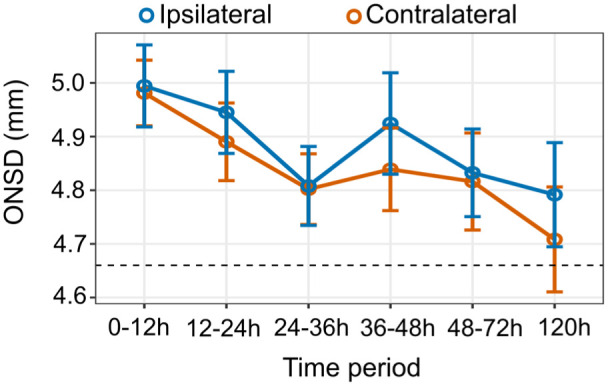
Longitudinal ONSDint measurements after stroke. Mean ONSDint is plotted for the ipsilateral and contralateral eye at six time points after stroke onset. Error bars represent the standard error of the mean. The dashed horizontal line indicates the mean ONSDint of the non-stroke control group (4.66 mm).

Serial ONSDint measurements were performed in stroke patients at up to six time points (Figure 3). The mean ONSDint was highest within the first 12 h after stroke onset (ipsilateral: 4.99 ± 0.56 mm; contralateral: 4.98 ± 0.45 mm; *n* = 53). The values remained elevated compared to the control group (4.66 ± 0.46 mm) throughout the observation period. A detailed summary of the measurements at each time point is provided in Supplemental Table 3.

A key finding was that there was no statistically significant difference between the ipsilateral and contralateral ONSDint at any of the measured time points (all *p* > 0.05, paired *t*-test). Therefore, for all subsequent analyses, the mean ONSDint of both eyes was used for each patient.

### ONSDint increases with grade of cerebral edema

When analyzing the initial ONSDint values (0–12 h) based on the radiological grade of cerebral edema, we observed a clear stepwise increase. Patients with no edema had a mean ONSDint of 4.91 ± 0.38 mm (*n* = 39), which increased in patients with mild-to-moderate edema to 5.03 ± 0.46 mm (*n* = 8). The highest initial values were observed in patients with severe edema who met the primary endpoint of IH, with a mean ONSDint of 5.43 ± 0.68 mm (*n* = 6).

**Figure 4.**
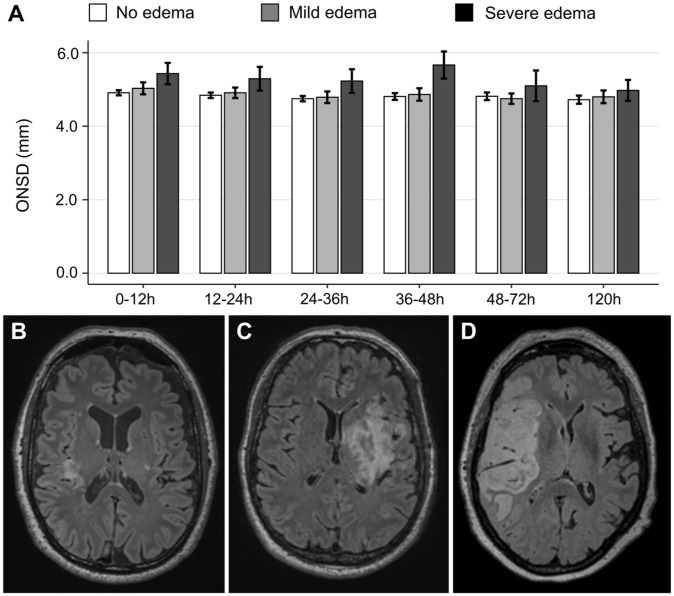
Longitudinal ONSDint by grade of cerebral edema: (a) mean ONSDint at six time points after stroke onset for patients with no edema (white bars), mild-to-moderate edema (gray bars), and severe edema (dark gray bars). Error bars represent the standard error of the mean, (b–d) representative MRI FLAIR images of a patient with no edema (b), mild-to-moderate edema (c), and severe edema with midline shift (d).

This pattern persisted over the entire observation period, with the severe edema group consistently showing the highest mean ONSDint values at each time point (Figure 4). Detailed longitudinal data for all edema groups are provided in Supplemental Table 4.

### Predictive value of sonographic and neuroimaging-derived ONSD

**Figure 5.**
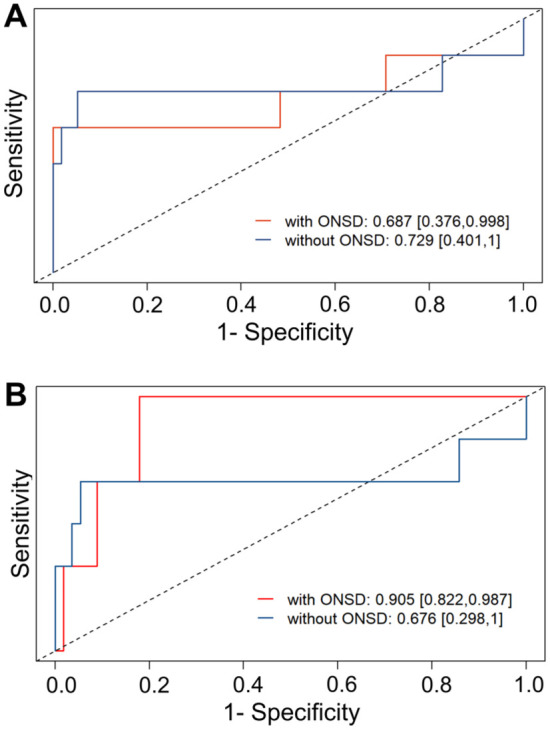
Predictive performance of ONSD for intracranial hypertension. Receiver Operating Characteristic (ROC) curves from the leave-one-out cross-validation. (a) The model including sonography-derived ONSDint (red line) compared to the baseline model without ONSD (blue line). (b) The model including CT/MRI-derived ONSD (red line) showed a substantial improvement in predictive performance compared to the baseline model. AUC values are provided with 95% confidence intervals.

To assess the predictive value of early ONSD measurement, we developed multivariable logistic regression models. Adding the initial sonographic ONSDint (measured within 12 h) to a baseline model containing age and infarct volume significantly improved the model fit (likelihood-ratio test, *p* = 0.012). From this model, an optimal cut-off value of 5.51 mm for sonographic ONSDint was determined, which predicted IH with a sensitivity of 85.7% and a specificity of 94.8%. In a leave-one-out cross-validation, the area under the curve (AUC) for the model including sonographic ONSDint was 0.687, compared to 0.729 for the baseline model (Figure 5).

We then performed a separate analysis using ONSD values derived from the initial CT or MRI scans, which were available for 62 of the 65 stroke patients. In the multivariable model, CT/MRI-derived ONSD was also a strong predictor of IH. The optimal cut-off value was 6.80 mm, which yielded a sensitivity of 100% and a specificity of 91.1%. In the cross-validation, the model including CT/MRI-derived ONSD achieved a substantially higher AUC of 0.905 (Figure 5). Detailed results of all regression models are provided in Supplemental Table 5.

## Discussion

In this prospective study, we demonstrate that early ONSD measurement is a valuable tool for predicting intracranial hypertension in patients with severe ischemic stroke. Our key finding is that sonographic ONSDint, measured within 12 h of symptom onset according to a standardized protocol, is a significant predictor of IH. After adjusting for age and infarct volume, we identified an optimal predictive cut-off of 5.51 mm, which classified high-risk patients with high sensitivity (86%) and specificity (95%).

Furthermore, we confirmed that ONSD measured on initial neuroimaging is also a powerful predictor for relevant IH. While the sonographic measurement provides a crucial bedside tool, the CT/MRI-derived ONSD showed a superior predictive performance in our cohort, with an AUC of 0.905 in the cross-validation.

Our sonographic ONSDint cut-off of 5.51 mm aligns well with previous studies in stroke patients that likely used a similar measurement technique, where thresholds of 5.4 and 5.6 mm were reported.^21,22^ This underscores the critical importance of standardized measurement protocols, as methodological differences, specifically the measurement of internal versus external ONSD, are a major source of heterogeneity in the literature, as highlighted by recent consensus criteria.^18^

A recent large, multi-center study by Zhang et al.^16^ strongly corroborates our finding that ONSD is a valuable biomarker in large hemispheric infarction. In their cohort of 419 patients, they found sonographic ONSD to be a powerful independent predictor of 90-day mortality. Interestingly, their study deliberately excluded patients receiving recanalization therapies, resulting in a very high mortality rate. Our study complements this work by demonstrating that ONSD is also a valuable predictor for the development of intracranial hypertension, a key mechanism leading to poor outcomes, specifically within a cohort of patients who have largely undergone reperfusion treatments. This suggests that the predictive value of ONSD is robust across both treated and untreated stroke populations, albeit for different but related endpoints. The utility of ONSD extends beyond ischemic stroke, as it has also been shown to hold prognostic value in other acute non-traumatic brain injuries, such as hypoxic-ischemic encephalopathy after cardiac arrest.^23^

A key finding was the symmetrical increase in ONSD, with no significant difference between the eye on the affected and unaffected hemisphere. This bilateral response underscores the principle that ONSD acts as a surrogate for global, rather than focal, intracranial pressure. This is crucial for its clinical utility in monitoring patients with large hemispheric strokes, as it indicates a generalized rise in pressure requiring intervention.

An important finding of our study was the direct comparison between sonographic and neuroimaging-derived ONSD. While our standardized sonography protocol yielded a clinically useful predictive threshold, the ONSD measured on the initial CT or MRI demonstrated superior predictive accuracy in our cross-validation analysis (AUC 0.905 vs 0.687). This difference may be attributable to the higher spatial resolution and lower operator dependency of CT and MRI. However, the unique and crucial advantage of transorbital sonography remains its utility as a non-invasive, repeatable, bedside tool that can be used for serial monitoring, which is not feasible with repeated CT or MRI scans.

Our CT/MRI-derived ONSD cut-off of 6.8 mm is notably higher than the 5.5 mm threshold reported in a similar study by Guo et al.^24^ A key difference that likely explains this discrepancy is the timing of the neuroimaging. In our study, the scans were performed in the hyperacute phase (median 2 h after onset), whereas in the study by Guo et al., imaging was conducted much later (mean 22 h after onset). This suggests that the predictive value of ONSD on structural imaging may be time-dependent, reflecting the dynamic evolution of cytotoxic edema.

To address the clinical integration of our findings, it is important to consider how early ONSD assessment could alter patient management. While not a substitute for comprehensive clinical or radiological assessment, ONSD could serve as a valuable, non-invasive adjunct for risk stratification. An elevated ONSD upon initial examination could trigger specific clinical pathways. For instance, it might prompt an increase in the frequency of neurological examinations or lead to a decision for earlier radiological follow-up to closely monitor edema progression. For high-risk patients, it could support the decision for prolonged monitoring in a specialized setting like a stroke unit. In centers without on-site neurosurgical facilities, a significantly elevated ONSD could also be a critical factor in the decision for prompt transfer to a comprehensive stroke center offering surgical decompression. By facilitating earlier risk stratification, these measures could help ensure timely and appropriate intervention.

Our study has several strengths that enhance the reliability of its findings. A key feature is the measurement of all sonographic data according to the latest international consensus criteria for ONSD measurement, ensuring high methodological quality and comparability. The prospective study design, inclusion of a non-stroke patient control group, and an early measurement window within 12 h of stroke onset are further strengths. Finally, by developing a multivariable predictive model and directly comparing sonography-derived ONSD with neuroimaging-derived values, our study provides a comprehensive assessment of this biomarker’s potential in the acute clinical setting.

Several limitations of our study must be acknowledged. The most significant is the small number of patients who developed our primary endpoint of intracranial hypertension (*n* = 7), which limits the statistical power of our predictive models. For this reason and to avoid the risk of overfitting, we refrained from including other important predictors of IH such as baseline NIHSS or recanalization status into our final model. Consequently, our findings, particularly the derived ONSD cut-off value, should be considered exploratory and hypothesis-generating. Similarly, a meaningful analysis of dynamic ONSD trends over time was precluded by this small subgroup size. Other limitations include some missing ONSD values due to the logistical challenges of serial measurements in an acute clinical setting, and the fact that our definition of IH was based on clinical and radiological surrogate markers rather than invasive ICP monitoring, the clinical gold standard. Therefore, validation of our predictive model in larger, multi-center prospective cohorts using standardized protocols is essential before these results can be implemented in routine clinical practice.

In conclusion, our study demonstrates that early, non-invasive ONSD measurement is a valuable predictor of intracranial hypertension after severe ischemic stroke. Using a standardized sonography protocol, we identified a predictive ONSDint cut-off of 5.51 mm, which classified high-risk patients with excellent sensitivity and specificity. While ONSD measured on initial neuroimaging showed superior predictive accuracy in our cohort, bedside transorbital sonography remains a crucial, repeatable tool for monitoring these critically ill patients. Thus, ONSD assessment can facilitate the early detection of high-risk patients, allowing for closer monitoring and timely intervention.

## Supplemental Material

sj-docx-1-eso-10.1177_23969873251379985 – Supplemental material for Optic nerve sheath diameter for prediction of intracranial hypertension after ischemic sTrokE – The ONSITE study Supplemental material, sj-docx-1-eso-10.1177_23969873251379985 for Optic nerve sheath diameter for prediction of intracranial hypertension after ischemic sTrokE – The ONSITE study by Philipp Baumgartner, Malin Zahn, Hannah-Lea Handelsmann, Kevin Geier, Sara Petrus, Martin Hänsel, Konstantin Mayr, Theodor Pipping, Andreas R Luft, Lisa Herzog and Susanne Wegener in European Stroke Journal
