# Supplementary material for: Optic nerve sheath diameter for prediction of intracranial hypertension after ischemic sTrokE – The ONSITE study
Source: Eur Stroke J. 2025 Sep 29:23969873251379985. Online ahead of print. doi: 10.1177/23969873251379985 (PMC12484050; doi:10.1177/23969873251379985)
Supplement: sj-docx-1-eso-10.1177_23969873251379985 – Supplemental material for Optic nerve sheath diameter for prediction of intracranial hypertension after ischemic sTrokE – The ONSITE study [file sj-docx-1-eso-10.1177_23969873251379985.docx]

**Supplementary Material**

| **Diagnosis** | **Number of controls (%)** |
| --- | --- |
| Parkinson’s disease | 10 (33) |
| Transient ischemic attack | 8 (27) |
| Cerebral atherosclerosis | 5 (17) |
| Peripheral nerve disease | 1 (3) |
| Gait ataxia | 1 (3) |
| Essential tremor | 1 (3) |
| Transient global amnesia | 1 (3) |
| Vestibular neuritis | 1 (3) |
| Multiple Sclerosis | 1 (3) |
| Transient monoocular vision loss | 1 (3) |

**Supplemental Table 1 Legend:** Main diagnosis at time of study participation in control patients

|  | **IH**  **n=7** | **Non-IH n=58** |
| --- | --- | --- |
| Age, median (IQR) | 61 (12) | 68 (14) |
| Sex, female (%) | 4 (57) | 22 (38) |
| BMI, median (IQR) | 23 (7) | 26 (7) |
| Atrial fibrillation (%) | 1 (14) | 17 (29) |
| Diabetes (%) | 1 (14) | 8 (14) |
| Arterial hypertension (%) | 2 (29) | 36 (62) |
| Dyslipidemia (%) | 1 (14) | 13 (22) |
| Current Smoker (%) | 3 (43) | 9 (16) |
| Prior ischemic stroke (%) | 0 (0) | 8 (14) |
| NIHSS on admission, median (IQR) | 18 (3) | 8 (9) |
| GCS on admission, median (IQR) | 13 (3) | 14 (3) |
| Large Vessel Occlusion (%) | 7 (100) | 40 (69) |
| Type of Occlusion |  |  |
| ICA (%) | 2 (29) | 11 (19) |
| MCA-M1 (%) | 5 (71) | 23 (40) |
| Tandem occlusion (%) | 0 (0) | 6 (10) |
| Acute Treatment |  |  |
| IVT Only (%) | 0 (0) | 11 (19) |
| EVT Only (%) | 3 (43) | 21 (38) |
| Combination (%) | 4 (57) | 20 (34) |
| Hemicraniectomy (%) | 2 (29) | 0 (0) |

**Supplemental Table 2 Legend: Baseline characteristics of patients developing IH vs those that did not develop IH.** BMI = body mass index, NIHSS = National Institutes of Health Stroke Scale, GCS = Glasgow Coma Scale, ICA = internal carotid artery, IH = Intracranial hypertension, MCA-M1 = middle cerebral artery M1 segment, IVT = intravenous thrombolysis, EVT = endovascular treatment

| **Timepoint after Stroke** | **Side** | **Mean ONSD (mm)** | **Standard derivation** | **n** |
| --- | --- | --- | --- | --- |
| 0-12h | Ipsilateral | 4.99 | 0.56 | 53 |
| 0-12h | Contralateral | 4.98 | 0.45 | 53 |
| 12-24h | Ipsilateral | 4.95 | 0.55 | 51 |
| 12-24h | Contralateral | 4.89 | 0.52 | 51 |
| 24-36h | Ipsilateral | 4.81 | 0.52 | 50 |
| 24-36h | Contralateral | 4.80 | 0.47 | 50 |
| 36-48h | Ipsilateral | 4.92 | 0.60 | 41 |
| 36-48h | Contralateral | 4.84 | 0.49 | 41 |
| 48-72h | Ipsilateral | 4.83 | 0.50 | 37 |
| 48-72h | Contralateral | 4.82 | 0.55 | 37 |
| 72-120h | Ipsilateral | 4.79 | 0.48 | 24 |
| 72-120h | Contralateral | 4.71 | 0.48 | 24 |

**Supplemental Table 3 Legend: Longitudinal ONSDint Measurements in patients after ischemic stroke** Data are presented as mean ± standard deviation for the ipsilateral and contralateral eye at each time point after stroke onset. N indicates the number of patients measured at each interval.

| **Timepoint after Stroke** | **Edema** | **Mean ONSD (mm)** | **Standard derivation** | **n** |
| --- | --- | --- | --- | --- |
| 0-12h | No edema | 4.91 | 0.44 | 39 |
| 0-12h | Mild-to-moderate | 5.03 | 0.46 | 8 |
| 0-12h | Severe | 5.43 | 0.71 | 6 |
| 12-24h | No edema | 4.84 | 0.43 | 34 |
| 12-24h | Mild-to-moderate | 4.91 | 0.45 | 10 |
| 12-24h | Severe | 5.29 | 0.85 | 7 |
| 24-36h | No edema | 4.75 | 0.43 | 36 |
| 24-36h | Mild-to-moderate | 4.79 | 0.48 | 9 |
| 24-36h | Severe | 5.23 | 0.71 | 5 |
| 36-48h | No edema | 4.81 | 0.51 | 31 |
| 36-48h | Mild-to-moderate | 4.86 | 0.45 | 7 |
| 36-48h | Severe | 5.67 | 0.64 | 3 |
| 48-72h | No edema | 4.82 | 0.53 | 26 |
| 48-72h | Mild-to-moderate | 4.75 | 0.40 | 8 |
| 48-72h | Severe | 5.1 | 0.72 | 3 |
| 72-120h | No edema | 4.72 | 0.50 | 20 |
| 72-120h | Mild-to-moderate | 4.8 | 0.25 | 2 |
| 72-120h | Severe | 4.97 | 0.40 | 2 |

**Supplemental Table 4 Legend: Longitudinal ONSDint Measurements by Grade of Cerebral Edema** Data are presented as mean ± standard deviation of the ONSDint (mm) for each radiological edema grade at six time points after stroke onset. N indicates the number of patients with available measurements in each group at each interval.

| **Sonographic ONSD** | | | |
| --- | --- | --- | --- |
|  | Log odds | Std. Error | Pr(>\|z\|) |
| Age | -0.7142 | 0.7357 | 0.3316 |
| Infarct volume | 1.3835 | 0.5581 | 0.0132 |
| ONSD | 1.4415 | 0.6878 | 0.0361 |
| **CT / MRI derived ONSD** | | | |
|  | Log odds | Std. Error | Pr(>\|z\|) |
| Age | -0.7128 | 0.7017 | 0.30971 |
| Infarct volume | 1.7007 | 0.6490 | 0.00878 |
| ONSD | 2.2686 | 1.0245 | 0.02680 |

**Supplemental Table 5 Legend: Logistic regression model for sonographic and CT / MRI derived values**. The log odds for IH are increased 1.44-fold if ONSD increases by one standard deviation. When infarct volume increases by one standard deviation, we have a 1.31-fold increase in the odds for developing IH.

**[insert Figure S1]**

**Supplemental Figure 1: ONSDint measurements in control subjects.** The plot shows the distribution of individual ONSDint measurements for the left and right eye, as assessed by two independent investigators in the 30 control subjects. Horizontal black lines represent the mean value for each group, and error bars indicate the standard deviation.
